# Effect of Dietary *Bacillus licheniformis* Supplementation on Growth Performance and Microbiota Diversity of Pekin Ducks

**DOI:** 10.3389/fvets.2022.832141

**Published:** 2022-02-21

**Authors:** Lei Li, Xueze lv, Xu Han, Chenglei Sun, Keying An, Wenwen Gao, Zhaofei Xia

**Affiliations:** ^1^College of Veterinary Medicine, China Agricultural University, Beijing, China; ^2^Beijing General Station of Animal Husbandry, Beijing, China

**Keywords:** *Bacillus licheniformis*, Pekin duck, metabolome, intestinal microbial, growth

## Abstract

This experiment was conducted to investigate the effects of different concentrations of *Bacillus licheniformis (B. licheniformis)* on growth performance and microbiota diversity of Pekin ducks. Three hundred 1-day-old healthy Pekin ducks were randomly divided into 5 groups with 6 replicates per group and 10 ducks per replicate. The five treatments supplemented with basal diets containing: either 0 (group CON), 200 (group LLB), 400 (group MLB), and 800 (group HLB) mg/kg *B. licheniformis* or 150 mg/kg aureomycin (group ANT) for 42 days, respectively, and were sacrificed and sampled in the morning of the 42nd day for detection of relevant indexes. The results showed as follows: The feed conversion ratio of the LLB group and MLB groups were lower than the CON group (*P* < 0.05). The body weight and average daily feed intake of the MLB group were significantly higher than that of the CON group and ANT group (*P* < 0.05). Compared with the CON group, the MLB group significantly increased the content of IgA (*P* < 0.05) and proinflammatory IL-6 were significantly decreased (*P* < 0.05), besides, the activity of SOD and T-AOC were also significantly increased in the MLB group (*P* < 0.05). The 16S rRNA analysis showed that *B. licheniformis* treatments had no effect (*P* > 0.05) on the alpha diversities of the intestine. The addition of *B. licheniformis* had a dynamic effect on the abundance of cecal microflora of Pekin ducks, and 1-21 d increased the diversity of microflora, while 21d-42 d decreased it. Compared with the CON group, the relative abundance of *Epsilonbacteraeota* in the MLB group was significantly increased on Day 21 (*P* < 0.05), and that of *Tenericutes* in the LLB group was significantly increased as well (*P* < 0.05). At 42 d, the relative abundance of *Bacteroidetes* in LLB, MBL, HBL, and ANT groups was significantly increased (*P* < 0.05). In addition, the addition of *B. licheniformis* increased the amount of SCAF-producing bacteria in the intestinal microbiota, such as *Lachnospiraceae, Collinsella, Christensenellaceae, and Bilophila*. The PICRUSt method was used to predict the intestinal microbiota function, and it was found that lipid transport and metabolism of intestinal microbiota in the MLB group were significantly affected. Overall, these results suggest diet supplemented with *B. licheniformis* improved growth performance, immune status, antioxidant capacity, and modulated intestinal microbiota in Pekin ducks. The optimal dietary supplement dose is 400 mg/kg.

## Introduction

Antibiotics have been widely used for many years to inhibit the pathogenicity of pathogens and promote the growth and development of animals. The use of antibiotics not only affects the target pathogen, but also benefits the gut microbiome, resulting in disease-related changes in the gut microbiome during growth (1). But in the process of antibiotic use, its side effects are constantly found, including antimicrobial resistance (AMR), antibiotic residues in food, animal products, and drug environmental pollution problems, has been a serious threat to animals and human health (2). For this reason, many countries, including China, have restricted the use of in-feed antibiotics (3, 4). With strict bans on antibiotics, there is a growing interest in finding green and safe alternatives to antibiotics in food animal production. In recent years, the application of probiotics in human, aquaculture, poultry, and livestock pathogen infection, as well as the regulation of host immune system, has aroused great interest (5).

*Bacillus licheniformis* is a gram-positive bacterium with high pathogenicity and temperature resistance. *B. licheniformis* can enhance the growth performance of chickens and maintain intestinal microbiota balance in broilers (6, 7). Previous studies have found that the *B. licheniformis* can produce various bioactive substances, such as digestive enzymes, lysozyme, bacteriocin, and antibacterial peptide, these substances through increased digestibility of feed, stimulate the immune system development, strengthen the function of intestinal mucosa to improve animal performance, disease-causing bacteria colonize, promote the potential beneficial microbial proliferation, and maintain the balance of intestinal microbiota (8–10). In addition, the diet supplemented with *B. licheniformis* can also regulate the composition and structure of intestinal microbiota of NE-stimulated broilers (11, 12). In the post-antibiotic era, it is urgent to develop green feed additives for healthy duck breeding in Beijing. The effects of *B. licheniformis* on Pekin ducks were affected by the number of viable bacteria and the stage of application, and the mechanism of growth promotion and immunity improvement was not exact, which needs to be further explored.

Therefore, the present study focused on the effects of *B. licheniformis* preparation with a different amount on growth performance, antioxidant indexes, and blood biochemical indexes of Pekin ducks, and studied the effects of *B. licheniformis* preparation with different concentration on cecal microbial diversity and bacterial community structure of Pekin ducks at a different time.

## Materials and Methods

All animal procedures were carried out in accordance with the Guidelines for Care and Use of Laboratory Animals of China Agricultural University, and the experimental program was approved by the Animal Care and Use Committee of China Agricultural University (Beijing, China).

### Experimental Design and Feeding Management

Three hundred 1-day-old healthy Pekin ducks with similar initial body weight were randomly divided into 5 groups with 6 replicates per group and 10 ducks per replicate. The CON group was fed a corn-soybean meal basal diet without antibiotics and growth promoting hormone. Experimental groups were fed the basal diet supplemented with 200 mg/kg *B. licheniformis* (LLB), 400 mg/kg *B. licheniformis* (MLB), 800 mg/kg *B. licheniformis* (HLB), and 150 mg/kg aureomycin (ANT), respectively. The basal diet was formulated according to NY/T2122-2012 Feeding Standard for Meat Ducks in China, and the composition and nutritional levels of the basal diet were shown in Table 1 (13). The probiotic strain *B. licheniformis* used in this study was purchased from Guangzhou Weiyuan Biotechnology Co., Ltd.

**Table 1:** Composition and nutrient levels of basal diets (air-dry basis, %).

**Items**	**1 to 21 d**	**22 to 42 d**
Ingredients		
Corn	56.00	60.24
Soybean meal	32.69	24.67
Wheat middling	5.00	9.00
Soybean oil	2.10	1.80
Phytases	0.02	0.02
CaHPO4	1.00	1.60
Limestone	1.50	1.20
DL-Met	0.15	0.12
L-Lys	0.20	0.10
Vitamin premix^a^	0.02	0.02
Trace mineral premix^b^	0.20	0.20
NaCl	0.35	0.30
Cholie chloride (50%)	0.24	0.20
Ethoxyquin (33%)	0.03	0.03
Maifanite	0.50	0.50
Total	100.00	100.00
Nutrient levels^c^		
ME	12.31	12.53
CP	19.52	16.83
Lys	1.12	0.87
Met	0.46	0.39
Ca	0.88	0.89
AP	0.29	0.39
TP	0.54	0.62
Met+Cys	0.79	0.69

a *The vitamin premix provided the following per kilogram of diet: vitamin A, 12,500 IU; vitamin D3, 3,500 IU; vitamin E, 20 IU; vitamin K3, 2.65 mg; thiamin, 2.00 mg; riboflavin, 6.00 mg; pyridoxin, 3.00 mg; VB12, 0.025 mg; biotin, 0.0325 mg; folic acid, 12.00 mg; pantothenic acid, 50 mg; nicotinic acid, 50.00 mg.*

b *The mineral premix provided the following per kilogram of diet: Cu, 6 mg; Fe, 80 mg; Zn, 40 mg; Mn, 100 mg; Se, 0.15 mg; I, 0.35 mg.*

c *The nutrient levels were calculated values*.

Before the formal test, the duck house should be cleaned and disinfected in all directions. The whole process of Pekin ducks was raised on net rearing, with no restriction on drinking water and food intake and 23 h of light. In the first week, the temperature of the duck house was maintained at 35°C, and then gradually reduced to room temperature of 25°C. Automatic temperature control equipment was used to control the temperature of the duck house. Immunization is carried out according to routine immunization procedures. Clean the feces at 7:00 am every day and clean the sink and trough at a weekly time. They are fed twice every day at 8:00 a.m. and 16:00 p.m. The experiment lasted for 42 days. All ducks were individually weighed (BW) and their feed intake was recorded at each replicate on Days 21 and 42, respectively. Average daily feed intake (ADFI), average daily gain (ADG), and feed conversion rate (FCR) were calculated.

### Sample Collection

At 8 a.m. of Day 21 and Day 42 of tests: At 00 (fasted 12 h in advance), 4 Pekin ducks were randomly selected from each replicate of each group for individual weighing and recording. Then 10 ml blood was collected from the jugular vein of the Pekin ducks, which was left standing at room temperature and taken back to the laboratory for centrifugation at 3,500 r/min for 15 min. The serum from the upper layer was separated and transferred to a new centrifugal tube. Stored in −20°C refrigerator for subsequent index detection, the cecal contents were taken out and placed in a 5 ml centrifuge tube, then cryopreserved with liquid nitrogen and stored in an −80°C ultra-low temperature refrigerator.

### Serum Content Analyses

The levels of serum IgA, IgM, C3, and C4 were determined using methods for ELISA commercial kits (Nanjing Jiancheng Bioengineering Co., Ltd., Nanjing, China). The concentrations of inflammatory cytokines IL-1β, IL-4, IL-6, IL-10 were quantified with duck special cytokine/chemokine kits (Nanjing Jiancheng Bioengineering Co., Ltd., Nanjing, China). GSH-Px, T-AOC, and SOD were used to evaluate the antioxidant level of the body, the contents of these indicators were determined with duck special cytokine/chemokine kits (Nanjing Jiancheng Bioengineering Co., Ltd., Nanjing, China). All measurements were conducted following the manufacturers' guidelines.

### Gut Microbiota Analysis

Microbial genomic DNA was extracted from cecal contents of broilers aged 21 and 42 d under sterile conditions using TGuide S96 fecal genomic DNA extraction reagent (Beijing Tiangen, China). The concentration of extracted nucleic acids was detected by using a microplate reader (GeneCompang Limited, Synergy HTX). According to the detection amplification, the PCR products were detected by electrophoresis with agarose at a concentration of 1.8% (Manufacturer: Beijing BMA Fuxin Technology Co., LTD.) to test the integrity. The specific primers 338F/806R (338F:5'-ACTCCTACGGGAGGCAGCA-3';806R:5'-GGACTACHVGGGTWTCTAAT−3') target fragments of amplified sample DNA. The mi-SEQ small fragment library was constructed with a DNA library construction kit, and the quality and concentration of the library were detected by Qubit 2.0 and Q-PCR. The amplified library was standardized, purified, and sequenced by Illumina HiSeq 2500 PE250 platform of Beijing BMB Biopharmaceutical Technology Co., LTD., (Shanghai, China).

### Statistical Analysis

Excel 2010 was used for input processing of test data, then IBM SPSS 26.0 analysis software was used for one-way ANOVA (LSD) of the mean value of test data, and Duncan's was used for post-multiple comparison test. Partial results were expressed in the format of “mean ± standard error”, where *P* < 0.05 represented significant differences in test results. Mothur (Version 1.35.0) software and R language tools were used to evaluate the sample Alpha diversity (Shannon, Simpson, ACE, Chao, and Coverage) (14). Variance analysis was used to determine differences in alpha diversity index. Principal coordinate analysis (PCoA) was conducted based on Bray Curtis distance of OTU relative abundance of cecal contents of Pekin ducks. The differences between groups were tested by similarity analysis (ANOSIM). Wilcoxon rank sum test was used to analyze the taxa differences at the phylum and genus levels.

## Results

### Growth Performance

The effects of *B. licheniformis* supplementation on growth performance of Pekin ducks are shown in Table 2. From Day 1 to 21, there were no significant differences in body weight, average daily gain, and feed ratio among 5 groups (*P* > 0.05), but the average daily feed intake of *B. licheniformis* supplemental group was significantly lower than that of the control group (*P* < 0.05). From 22 to 42 days, body weight, average daily gain, and feed conversion rate of different treatment groups had no significant difference. From 1 to 42 d, compared with the CON group, body weight, average daily gain, and feed ratio of Pekin ducks in the MLB and HLB groups were significantly increased (*P* < 0.05), and feed conversion rate of Pekin ducks in the LLB and MLB groups was decreased compared with the CON group. Meanwhile, the growth performance of Pekin ducks was not significantly improved by adding antibiotics (*P* > 0.05).

**Table 2:** Effects of *B. licheniformis* and aureomycin on growth performance of Pekin ducks^1^.

**Items**	**CON**	**LLB**	**MLB**	**HLB**	**ANT**	**SEM**	***P*-value**
**Body weight, kg**							
Day 1 to 21	1.16	1.15	1.16	1.14	1.14	0.014	0.11
Day 21 to 42	2.91	2.94	3.21	3.07	2.78	0.18	0.38
Day 1 to 42	2.91^a^	2.94^a^	3.21^b^	3.07^b^	2.78^a^	0.18	0.013
**Average daily gain, g/d**							
Day 1 to 21	55.16	54.88	55.14	55.35	54.49	0.67	0.11
Day 21 to 42	83.25	85.00	97.62	91.84	78.03	8.42	0.37
Day 1 to 42	69.21^a^	69.94^a^	76.38^b^	73.10^b^	66.26^a^	4.34	0.02
**Average daily feed intake, g/d**							
Day 1 to 21	95.67^a^	90.13^c^	93.55^b^	91.75^bc^	90.02^abc^	3.08	0.002
Day 21 to 42	245.37	237.07	271.71	266.06	228.36	20.19	0.46
Day 1 to 42	170.52^ac^	163.61^a^	182.63^b^	178.91^b^	159.19^c^	10.37	0.025
**Feed conversion rate**							
Day 1 to 21	1.73	1.64	1.70	1.69	1.65	0.06	0.09
Day 21 to 42	2.94	2.79	2.78	2.90	2.93	0.10	0.16
Day 1 to 42	2.46^a^	2.34^b^	2.39^b^	2.45^a^	2.40^ab^	0.07	0.018

1 *CON, control group, basal diet; LLB, basal diet+200 mg/kg B. licheniformis; MLB, basal diet + 400 mg/kg B. licheniformis; HLB, basal diet+800 mg/kg B. licheniformis; ANT, basal diet+150 mg/kg aureomycin. ^a, b, c^ Within a row, values with different superscripts indicate a significant difference (P < 0.05)*.

### Analysis of Serum Inflammatory Factors, Immune and Antioxidant Levels

**Figure 1:**
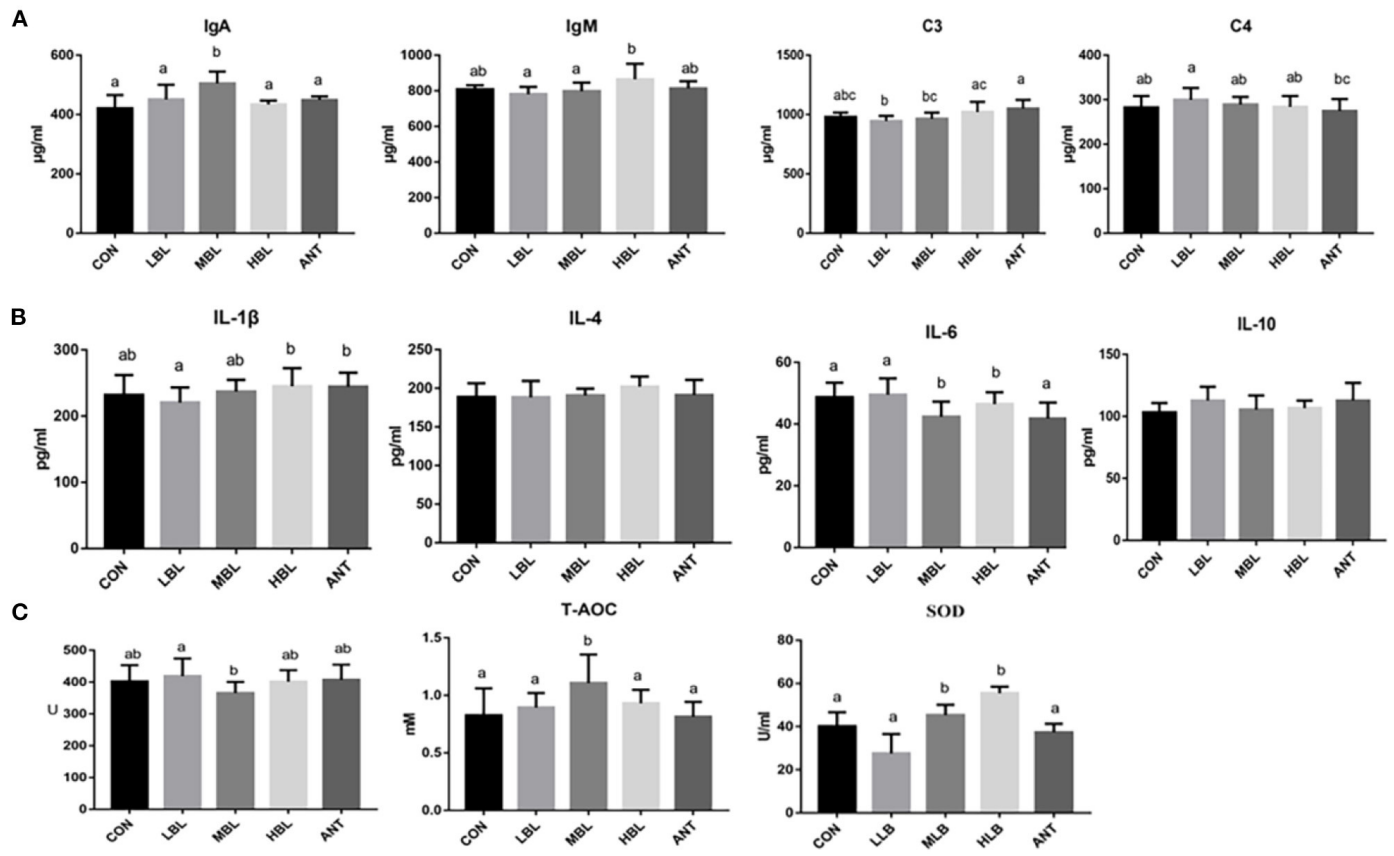
Effects of *B. licheniformis* on inflammatory factors, immune indices, and antioxidant indices of Pekin ducks. **(A)** immune indices; **(B)** inflammatory factors; **(C)** antioxidant indices. CON, control group, basal diet; LLB, basal diet+200 mg/kg *B. licheniformis*; MLB, basal diet+400 mg/kg *B. licheniformis*; HLB, basal diet+800 mg/kg *B. licheniformis*; ANT, basal diet+150 mg/kg aureomycin.

At 42 d, the content of immunoglobulin IgA of Pekin ducks in the MLB group was significantly increased compared with the CON group (*P* < 0.05), and the addition of any dose of *B. licheniformis* or antibiotics in the diet did not significantly affect the content of complement C3 and C4 (*P* > 0.05) shown in Figure 1A. As shown in Figure 1B, compared with the CON group, the contents of IL-4, IL-10, and IL-1β of Pekin ducks had no significant effect in the *B. licheniformis* groups (*P* > 0.05), but the content of IL-6 in the MLB and HLB groups was significantly decreased (*P* < 0.05). As shown in Figure 1C, compared with the CON group, SOD and T-AOC were significantly increased at 42 d (*P* < 0.05), but the supplementation of *B. licheniformis* and antibiotics had no significant difference in the content of GSH-Px of Pekin ducks (*P* > 0.05).

### Effect of *B. licheniformis* Supplementation on Sequence Data, Alpha-Diversity, and Beta-Diversity

After stringent quality trimming of raw data, the averages of high-quality reads from the cecal content of Pekin ducks in the CON group, LLB group, MLB group, HLB group, and ANT group were 66,395, 70,522, 66,477, 66,388 and 67,169 at 21 d, 63,841, 68,228, 68,451, 67,508, and 64,330 at 42 d. All effective tags of the cecal content samples of Pekin ducks were clustered into OTUs with 97% consistency. At 21 d and 42 d, OTU numbers of cecal microorganisms in each group fluctuated between 400 to 480, and there was no significant difference of OTUs number between groups, respectively. Intestinal microbiota plays crucial roles in maintaining gut homeostasis. Alpha diversity (Shannon and Simpson), richness estimators (Chao and ACE), and the coverage (good's coverage estimator) were used to investigate the effects of *B. licheniformis* on intestinal microbial abundance and diversity of Pekin ducks which are shown in Table 3. The results showed that *B. licheniformis* treatments had no effect (*P* > 0.05) on the alpha diversities of the intestinal microbiota in Pekin ducks at 21 d and 42 d (*P* > 0.05).

**Table 3:** Sequencing data and the alpha diversity in each group of Pekin ducks^1^.

**Items**	**CON**	**LLB**	**MLB**	**HLB**	**ANT**	**SEM**	***P*-value**
**Seq_num**							
Day 21	66,395.33^b^	70,522.50^a^	66,477.83^b^	66,388.8b^b^	67,169.17^b^	2523.14	0.010
Day 42	63,841.33	68,228.67	68,451.33	67,508.17	64,330.67	4585.20	0.253
**OTU_num**							
Day 21	424.50	402.83	412.83	430.50	410.50	21.73	0.190
Day 42	472.83	435.00	406.50	410.50	449.50	55.67	0.223
**ACE**							
Day 21	441.83	426.86	429.39	447.32	432.43	15.64	0.119
Day 42	499.18	459.46	442.89	449.18	479.13	46.08	0.213
**Chao**							
Day 21	446.92	429.32	439.22	449.16	438.78	16.34	0.260
Day 42	507.30	464.95	455.26	455.58	490.41	44.56	0.176
**Simpson**							
Day 21	0.9468	0.9415	0.9530	0.9587	0.9322	0.02	0.223
Day 42	0.9166	0.8862	0.8867	0.8968	0.9015	0.03	0.435
**Shannon**							
Day 21	5.81	5.58	5.83	5.07	5.45	0.39	0.157
Day 42	5.27	5.07	4.54	4.79	5.06	0.56	0.205
**Coverage**							
Day 21	0.9994	0.9994	0.9995	0.9995	0.9994	0.0001	0.636
Day 42	0.9993	0.9994	0.9992	0.9992	0.9992	0.0002	0.193

*Seq_num, sequence number; OTU_num, operational taxonomic unit number; ACE, abundance-based coverage estimator.*

1 *Pekin ducks were used as the experimental unit, n = 6 for each group. CON, control group, basal diet; LLB, basal diet+200 mg/kg B. licheniformis; MLB, basal diet +4 00 mg/kg B. licheniformis; HLB, basal diet+800 mg/kg B. licheniformis; ANT, basal diet+150 mg/kg aureomycin. When the main effect was significant or the interaction effect was significant, the minimum significant difference method was used to compare the mean values with a P < 0.05 indicating significance. a, b values with different superscripts indicate a significant difference (P < 0.05)*.

**Figure 2:**
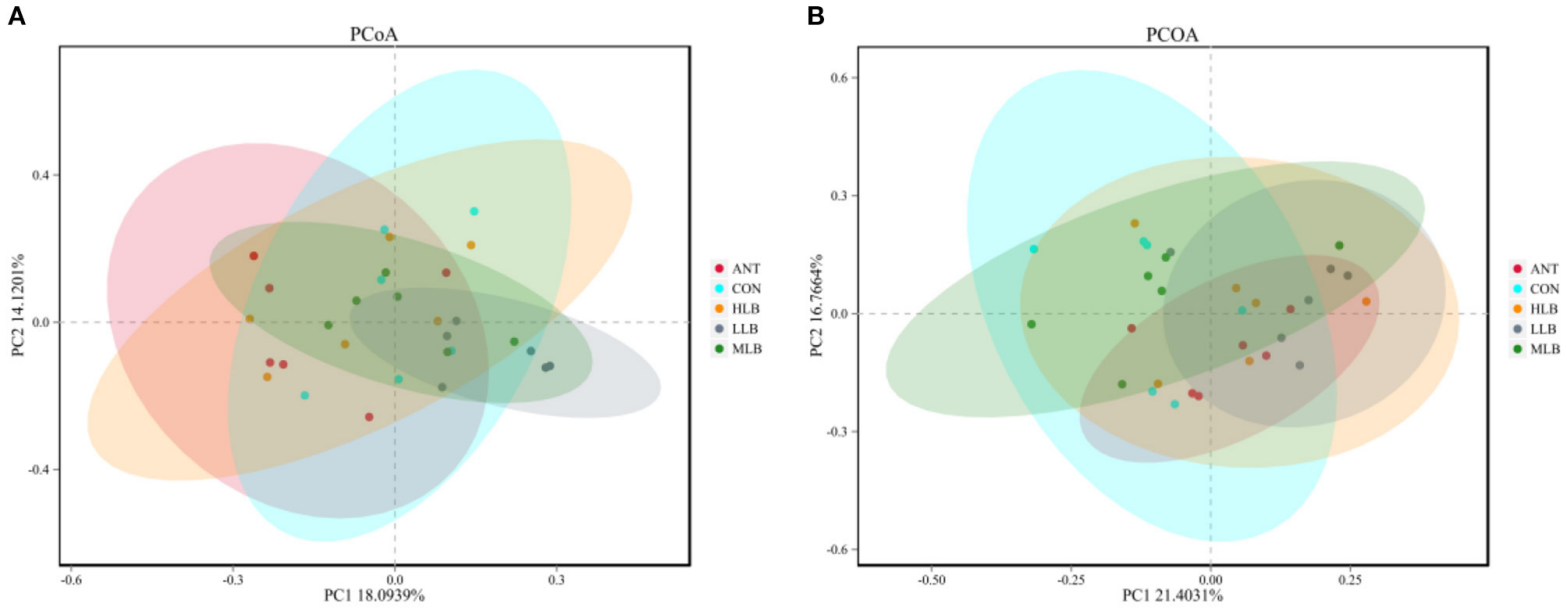
Principal coordinates analysis (PCoA) of microbial communities among groups based on Bray-Curtis distance, *n* = 6 for each group. CON, control group, basal diet; LLB, basal diet + 200 mg/kg *B. licheniformis*; MLB, basal diet+400 mg/kg *B. licheniformis*; HLB, basal diet+800 mg/kg *B. licheniformis*; ANT, basal diet+150 mg/kg aureomycin. **(A)** at 21d; **(B)** at 42d.

To assess overall differences in beta diversity, PCoA was used to identify differences among the 5 groups. Bray Curtis distance was used to analyze the relative abundance of OTU in the intestinal microflora of Pekin ducks which was further confirmed by ANOSIM and PERMANOVA analysis (Table 4). As shown in Figure 2A, at 21 d, the cecal microflora similarity of the CON group, LLB, MBL, HBL group, and ANT group was (ANOSIM: R = 0.241, *P* = 0.001), and as shown in Figure 2B, at 42 d, the cecal microbiota similarity of the CON group, LLB, MBL, HBL group, and ANT group was (ANOSIM: R = 0.224, *P* = 0.003), indicating that the difference between groups was greater than the difference within the group, and there were significant differences in bacterial communities among the five groups.

**Table 4:** ANOSIM and PERMANOVA analysis of microbial diversity among different treatments^1^.

**Item**	**ANOSIM**		**PERMANOVA**	
	***R***	***P-value***	***R^2^***	***P-value***
D 21				
Treatment	0.241	0.001	0.221	0.001
CON vs. LLB group	0.201	0.009	0.190	0.013
LLB vs. MLB vs. HLB	0.258	0.013	0.190	0.014
CON vs. MLB vs. ANT	0.195	0.009	0.176	0.015
D 42				
Treatment	0.224	0.003	0.228	0.0002
CON vs. LLB group	0.224	0.005	0.218	0.006
LLB vs. MLB vs. HLB	0.222	0.018	0.198	0.025
CON vs. MLB vs. ANT	0.244	0.010	0.196	0.015

*ANOSIM, analysis of similarities; PERMANOVA, permutational multivariate analysis of variance.*

1 *CON, control group, basal diet; LB group, LLB, MLB, and HLB; LLB, basal diet + 200 mg/kg B. licheniformis; MLB, basal diet + 400 mg/kg B. licheniformis; HLB, basal diet + 800 mg/kg B. licheniformis; ANT, basal diet+150 mg/kg aureomycin*.

### Effect of *B. licheniformis* Supplementation on Microbial Community Composition at the Phylum or Genus Level

**Figure 3:**
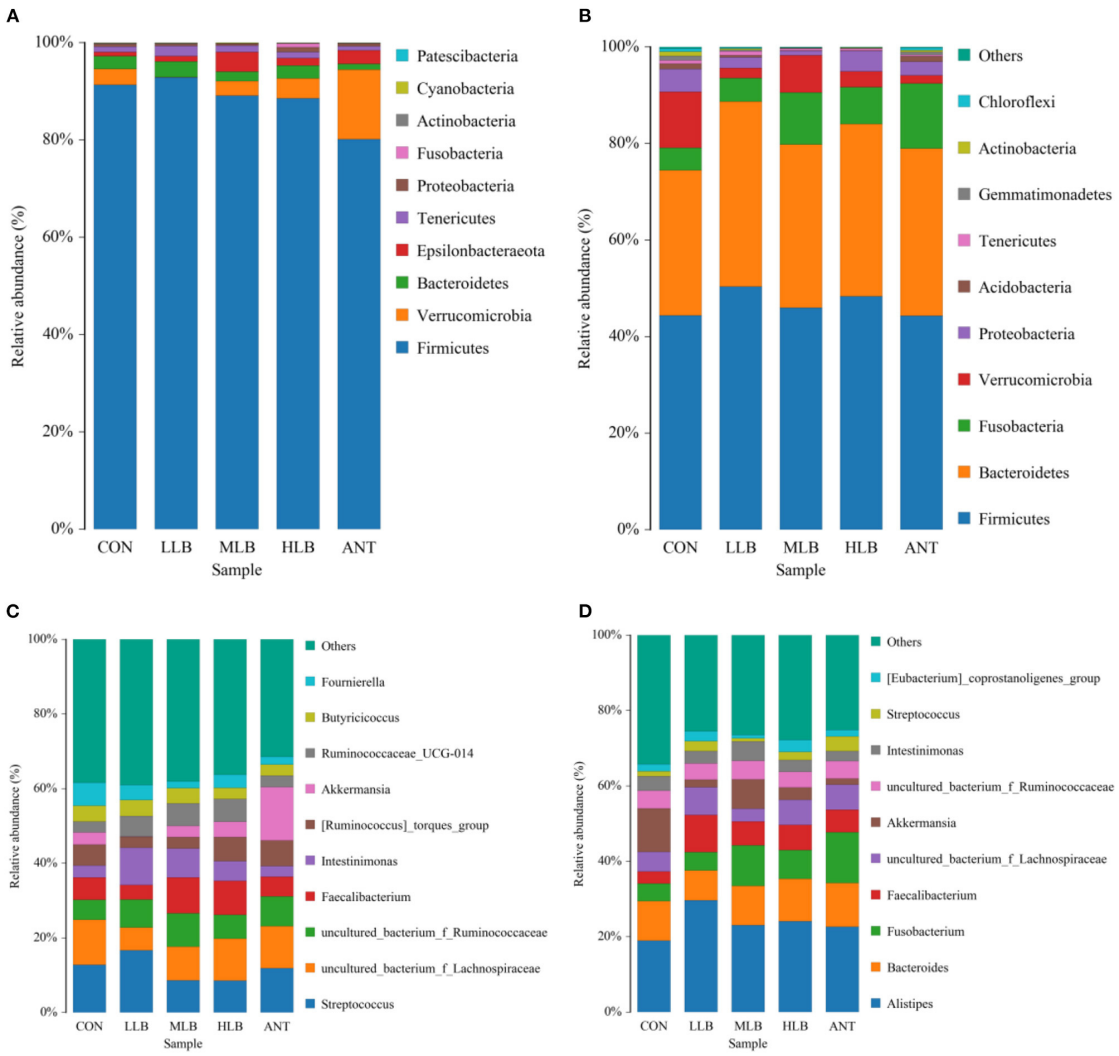
Relative abundance (%) and composition of intestinal microorganisms at the phylum level and the genus level at different time points of Pekin ducks, *n* = 6 for each group. **(A)** 21 d at phylum level; **(B)** 42 d at the phylum level; **(C)** 21 d at the genus level; and **(D)** 42 d at the genus level. CON, control group, basal diet; LLB, basal diet+200 mg/kg *B. licheniformis*; MLB, basal diet + 400 mg/kg *B. licheniformis*; HLB, basal diet + 800 mg/kg *B. licheniformis*; ANT, basal diet + 150 mg/kg aureomycin.

The relative abundance of bacteria at the phylum level of all samples at 21 d is shown in Figure 3A. As shown, 10 different phylum level classifications were identified, the top 5 dominant phyla with relative abundance >1% were *Firmicutes, Verrucomicrobia, Bacteroidetes, Epsilonbacteraeota*, and *Tenericutes*. *Firmicutes* was the most dominant bacteria in all the treatment groups, followed by *Verrucomicrophyla*. The relative abundance of *Firmicute*s in each group was 91.36% (CON), 92.83% (LLB), 89.10% (MLB), 88.57% (HLB), and 80.12% (ANT), respectively. The relative abundance of *Verrucomicrobia* in each group was 3.24% (CON), 6.22% (LCB), 2.98% (MCB), 4.11% (HCB), and 14.36% (ANT). The ratios of *Firmicutes* to *Verrucomicrobia* were 28.23 (CON), 14.94 (LCB), 29.86 (MCB), 21.54 (HCB), and 5.58 (ANT), respectively. At 42 d, a total of 10 different phylum level classifications were identified at the phylum level, among which there were 5 phylum level classifications with relative abundance >1% as shown in Figure 3B. They are *Firmicutes, Bacteroidetes, Fusobacteria, Verrucomicrobia*, and *Proteobacteria*. *Firmicutes* and *Bacteroidetes* were relatively dominant in all the treatment groups. The relative abundance of *Firmicutes* in each group was 44.39% (CON), 50.33% (LCB), 45.99% (MCB), 48.33% (HCB), and 44.33% (ANT), respectively. The relative abundance of *Bacteroidetes* increased significantly, and the relative abundance of *Bacteroidetes* in each group was 30.07% (CON), 38.38% (LCB), 33.78% (MCB), 35.73% (HCB), and 34.66% (ANT). The ratios of *Firmicutes*/*Bacteroidetes* were 1.48 (CON), 1.31 (LCB), 1.36 (MCB), 1.35 (HCB), and 1.28 (ANT), respectively.

As shown in Figure 3C, at 21 d, the top 10 genus level classifications with abundance level accounted for 60% of the overall level, and their abundance level was relatively average. *Streptococcus* was the dominant bacterium in all treatment groups, followed by *Fecalibacterium*. The relative abundance of *Streptococcus* in each group was 12.80% (CON), 16.65% (LLB), 8.63% (MLB), 8.58% (HLB), and 11.90% (ANT), respectively. The relative abundances of *Fecalibacterium* in each group are 5.91% (CON), 3.90% (LCB), 9.59% (MCB), 9.07% (HCB), and 5.25% (ANT), respectively. The ratios of *Streptococcus*/*Fecalibacterium* were 2.16 (CON), 4.26 (LCB), 0.90 (MCB), 0.95 (HCB), and 2.26 (ANT), respectively. As shown in Figure 3D, at 42 d, the top 10 genus level classifications in the abundance level accounted for 65% of the total level. *Alistipes* was the most dominant genus in all treatment groups, followed by *Bacteroides*. The relative abundance of *Alistipes* in each group was 18.99% (CON), 29.66% (LLB), 23.07% (MLB), 24.14% (HLB), and 22.68% (ANT), respectively. The relative abundance of *Bacteroides* in each group was 10.50% (CON), 7.90% (LCB), 10.42% (MCB), 11.24% (HCB), and 11.53% (ANT). The ratios of *Alistipes*/*Bacteroides* were 1.81 (CON), 3.75 (LCB), 2.21 (MCB), 2.15 (HCB), and 1.97 (ANT), respectively.

**Figure 4:**
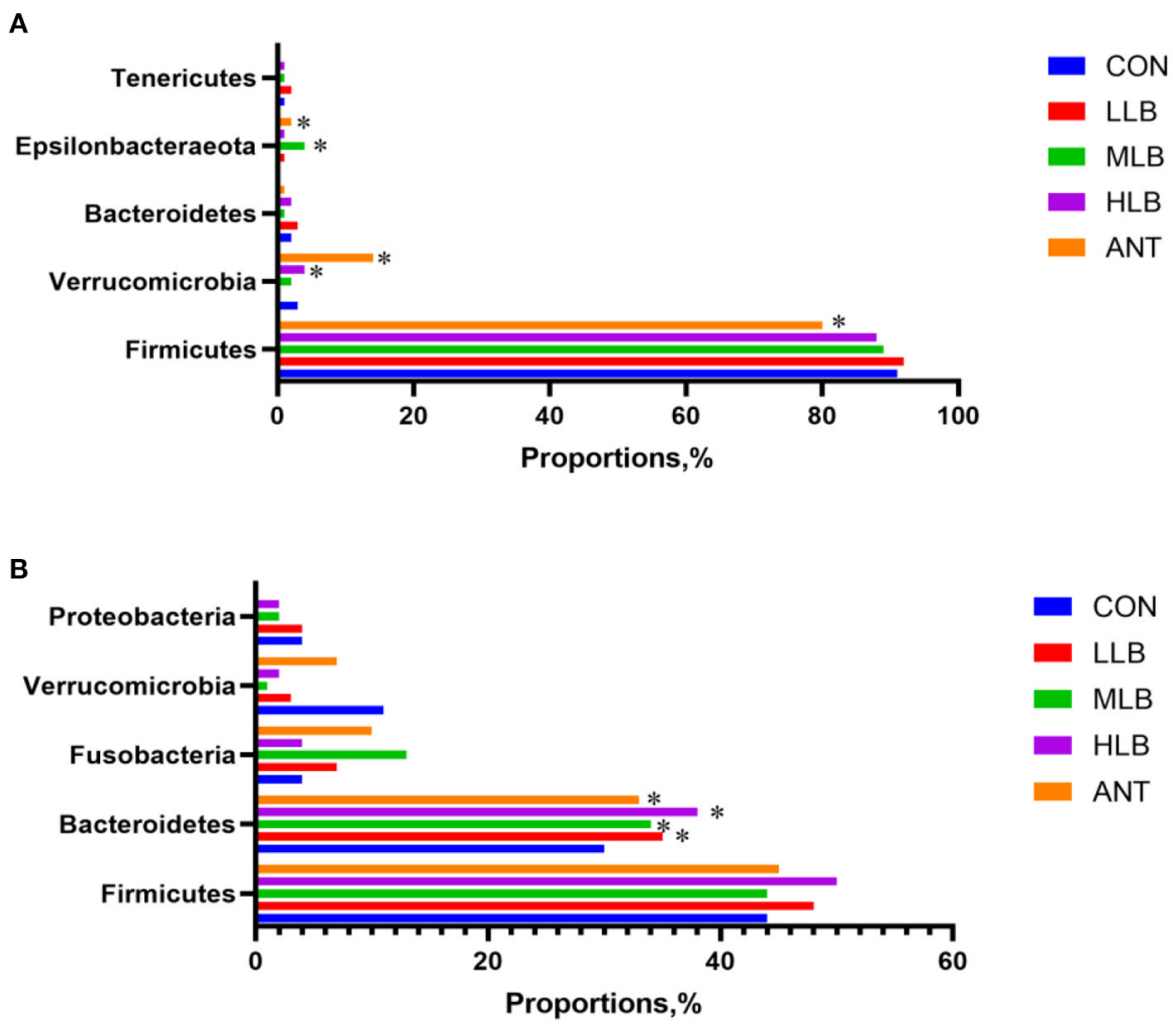
Differences of intestinal microbiota in experimental groups at the phylum level, *n* = 6 for each group. **(A)** 21 d at the phylum level; **(B)** 42 d at the phylum level. CON, control group, basal diet; LLB, basal diet + 200 mg/kg *B. licheniformis*; MLB, basal diet + 400 mg/kg *B. licheniformis*; HLB, basal diet+800 mg/kg *B. licheniformis*; ANT, basal diet+150 mg/kg aureomycin. * means significant difference compared with control (*P* < 0.05).

The differences of intestinal microbiota in experimental groups at the phylum level were analyzed in Figure 4. At 21 d, the results of intestinal bacterial composition showed that the relative abundance of *Tenericutes* in the LLB group was significantly (*P* < 0.05) increased, and the relative abundance of *Epsilonbacteraeota* in the MLB and ANT groups was significantly (*P* < 0.05) increased. But there was no significant difference in the relative abundance of *Firmicutes* among all groups (*P* > 0.05) in Figure 4A. At 42 d, compared with the CON group, the relative abundance of *Bacteroidetes* in the LLB group, MBL group, HBL group, and ANT group was significantly (*P* < 0.05) increased. In addition, there was no significant difference in the relative abundance of *Firmicutes* and *Proteobacteria* among all groups (*P* > 0.05) in Figure 4B.

### Analysis of Significant Differences Between Groups

**Figure 5:**
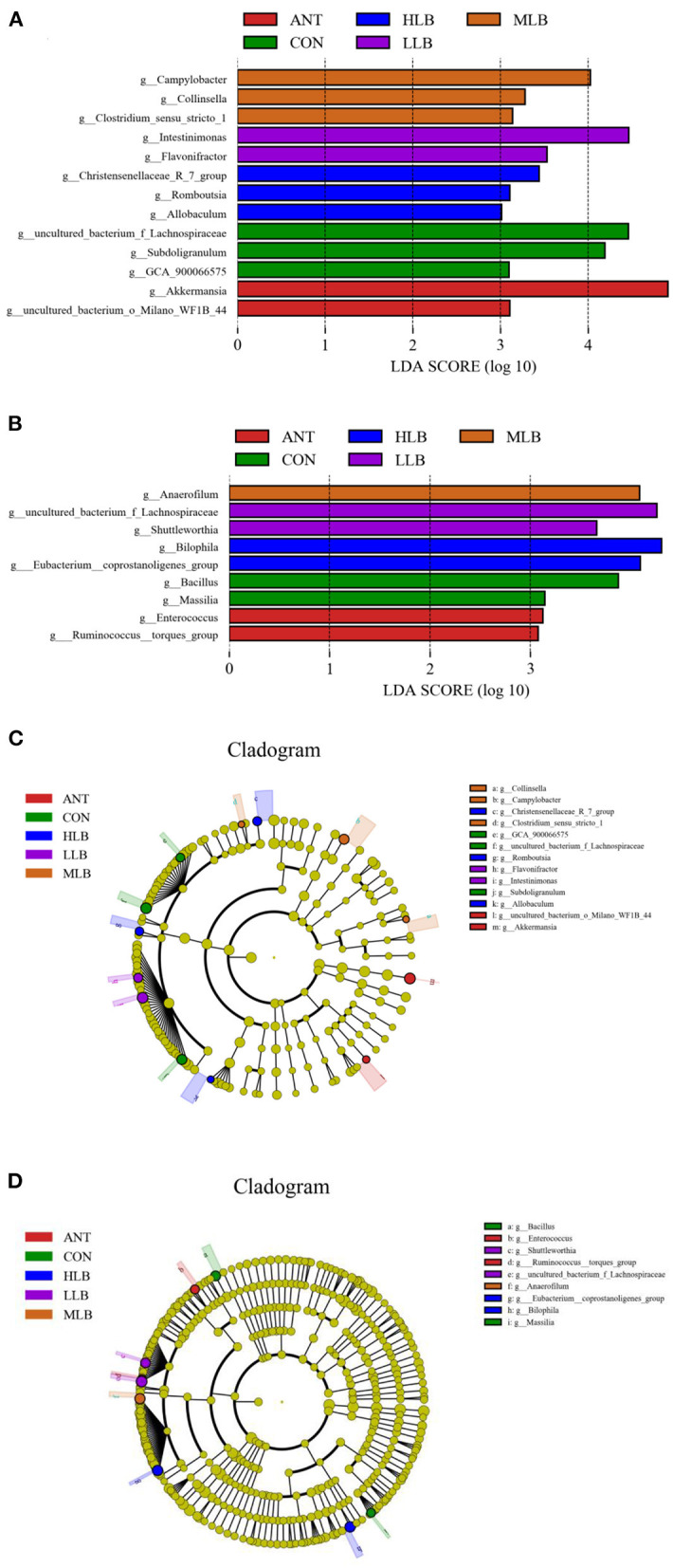
Histogram of LDA value distribution between different groups and LEfSe analysis evolutionary branching diagram, *n* = 6 for each group. Histogram of LDA values of intestinal microflora of Pekin ducks at 21 days **(A)** and 42 days **(B)** in the control group (CON) and the medium dose group (MLB). LEfSe analysis of evolutionary branching of intestinal microflora of Pekin duck at 21 **(C)** and 42 days **(D)** in the control (CON) and the medium dose (MLB) groups.

LEfSe (line discriminant analysis [LDA] effect size) was given the ability to find specific bacteria with statistical differences between groups with different concentrations of *B. licheniformis*. The non-parametric Kruskal-Wallis rank-sum test was used to detect species with significant differences in abundance between different groups first, and then the Wilcoxon rank-sum test was used for the consistency of differences between different subgroups of species in the previous step. Finally, LDA was used to estimate the impact of each component (species) abundance on the differential effect. As shown in Figure 5A, at 21 d, *lachnospiraceae* in the control group was significantly higher than that in the *B. licheniformis* addition group and the antibiotic group. *Flavonifractor, Intestinimonas, Campylobacter, Collinsella, Christensenellaceae*, and *Romboutsia* bacteria were a marked increase in relative abundance. The relative abundance of *Akkermansia* in the antibiotic group was significantly higher than that in other experimental groups. As shown in Figure 5B, at 42 d, the relative abundance of *Bacillus* and *Masillia* in the CON group was significantly higher than that in the *B. licheniformis* addition group and the antibiotic group, while the *B. licheniformis* addition group included *Lachnospiraceae, Ahuttleworthia*, and *Anaerofilum*, such as *Bilophila* relative abundance. The relative abundance of *Enterococcus* and *Ruminococcus* in the antibiotic group was significantly higher than that in the *B. licheniformis* addition group and CON group. Figures 5C,D are phylogenetic clades of different species. Circles radiating from inside to outside represent taxonomic levels from phylum to genus. Figure 5C showed that on 21d, *Lachnospiraceae* in CON group, the *Intestinimonas* in LLB group, *Campylobacter* in MLB group, *Christensenellaceae* in HLB group and *Akkermansia* in ANT group had the highest abundance in each group. As can be seen from Figure 5D, *Bacillus* in CON group, *Lachnospiraceae* in LLB group, *Anaerofilum* in MLB group, Bilophila in HLB group and *Enterococcus* in ANT group had the highest abundance in each group at 42d.

### Predictive Analysis of Functional Genes Among Samples

**Figure 6:**
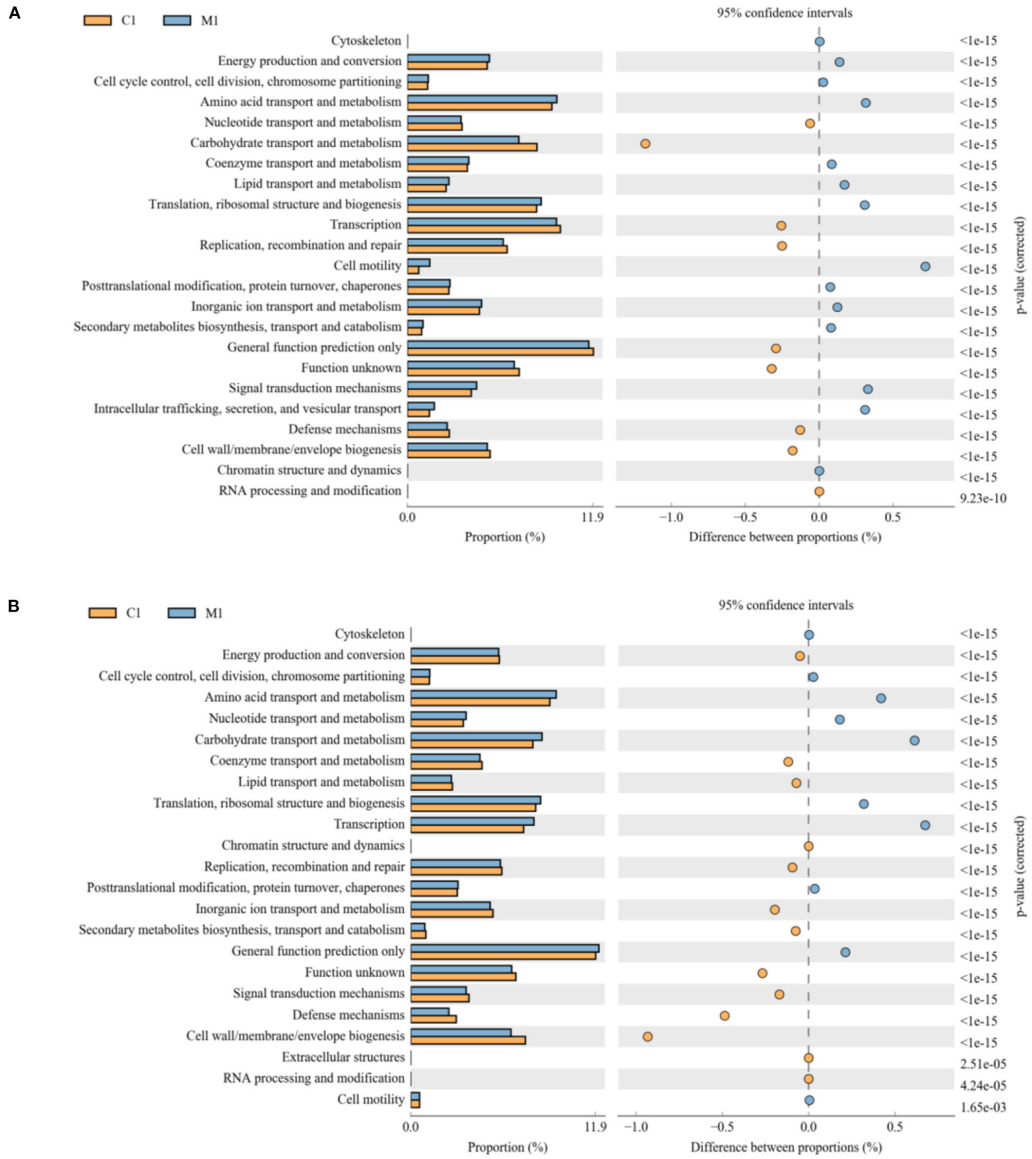
Comparison of predicted pathway abundances between the groups by statistical analysis of taxonomic and functional profiles (STAMP). **(A)** CON vs. MLB at 21 d; **(B)** CON vs. MLB at 42 d. CON, control group, basal diet; MLB, basal diet + 400 mg/kg *B. licheniformis*.

PICRUSt2 software was used to analyze the difference in function between different groups. COG (clusters of orthologous groups of proteins) function prediction reflected the functional distribution and abundance of sequences in the samples shown in Figure 6. At 21 d shown in Figure 6A, comparing with MLB, carbohydrate transport and metabolism, transcription, replication, general function prediction only, amino lipid transport and metabolism, and cell wall were enriched in the CON (*P* < 0.01), whereas cell motility, amino acid transport, ribosomal structure, lipid transport, signal transduction mechanisms, and intracellular trafficking were significantly enriched in MLB (*P* < 0.01).At 42 d shown in Figure 6B, comparing with MLB, amino lipid transport and metabolism, carbohydrate transport and metabolism, energy production and conversion, signal transduction mechanisms, secondary metabolites biosynthesis, inorganic ion transport, replication, lipid transport, and coenzyme transport were enriched in the CON (*P* < 0.01), meanwhile general function prediction only, transcription, translation, carbohydrate transport, nucleotide transport, and amino acid transport were significantly enriched in MLB (*P* < 0.01).

## Discussion

More and more studies have shown that probiotics have the functions of preventing intestinal infectious diseases, improving the production performance of poultry and improving the quality of poultry products, as a result, probiotics are considered as a green and safe alternative to antibiotics (15–17). The addition of probiotics in the diet can promote the growth of beneficial bacteria and ensure the healthier intestinal system, to improve the growth performance of broilers (18, 19). With the increase of research on probiotics, *B. licheniformis* has also appeared in the public eye (20). Previous studies have shown that *B. licheniformis* can improve the growth performance of chickens (6, 7). A previous study showed that the addition of *B. licheniformis* to drinking water effectively improved the growth performance of broilers (BW, ADG and FCR) (7). Another study found that adding *B. licheniformis* to the diet could increase BW and ADG of broilers (21). At the same time, there was a study showed that the addition of *Bacillus subtilis* and *B. licheniformis* in a broiler diet could increase BW, ADG, and ADFI and reduce the F: G ratio to improve growth performance at the beginning of feeding (22). Similar to the previous research results, in this experiment, the addition of 400 mg/kg *B. licheniformis* in the feed not only increased the final body weight and daily gain of Pekin ducks, but also reduced the feed meat ratio. In addition, other studies have shown that dietary supplementation with *B. licheniformis* can improve the growth performance of experimental animals even in the case of heat stress, immune stress, or necrotizing enteritis (23–27). The improvement of growth performance may be related to the beneficial metabolites produced by *B. licheniformis*, such as extracellular digestive enzymes, lysozymes, antifungal proteins, and various antibiotics (8, 28). It is also possible that the addition of *B. licheniformis* can enhance the immunity of broilers by regulating the composition and metabolic function of intestinal microbiota (6, 22, 29–31).

Studies have confirmed that probiotics can improve the resistance of livestock and poultry by strengthening their overall innate immunity (26). Serum immunoglobulins, especially IgA, IgG, and IgM produced by B cells, are important parameters reflecting the humoral immune status of animals, which are related to their important role in immune function and providing resistance to various infections (32–34). In previous studies, levels of IgA and IgM were elevated in chickens fed with *B. licheniformis* (35). In this study, the contents of IgA, IgM, C3, and C4 of *B. licheniformis* group were increased compared with the CON group. Among them, IgA in the MLB group was significantly higher than that in the CON group (*P* < 0.05). The results indicated that *B. licheniformis* could improve the immune level and enhance the disease resistance of Pekin ducks. Inflammatory factors play an important role in immune regulation when animals deal with various pathogenic bacteria infections. IL-6 is a powerful cytokine with a wide range of biological activities and can act on almost all cells of the immune system, playing a key role in regulating the host's immune response and hematopoietic function (36–38). Compared with the CON group, the serum IL-6 content in the MLB group was significantly decreased (*P* < 0.05). It indicates that *B. licheniformis* can improve the body's anti-inflammatory level to a certain extent, to better resist the infection of pathogenic bacteria. At present, studies have proved that some probiotics have good antioxidant capacity. On the one hand, they can produce enzyme forming catalysts such as GSH and SOD; on the other hand, they can reduce oxidative stress by changing the internal environment of the host intestinal tract (39). In this study, the activities of T-AOC and SOD of Pekin ducks supplemented with *B. licheniformis* were increased compared with those in the CON group, indicating that *B. licheniformis* can improve the activity of antioxidant enzymes and thus improve the antioxidant capacity of Pekin ducks.

The gut microbiome co-evolved with the host to form microorganisms with stable intestinal microenvironments that provide a wide range of biological functions for the host, such as digestion of complex dietary carbohydrates, production of absorbable nutrients and vitamins, resistance to pathogenic infections, and maintenance of intestinal environmental balance (40, 41). The cecum is the most abundant and concentrated intestinal microbiota. The biological fermentation process, especially the production of SCFA, is conducted in the cecum. In addition, the intestinal microbiota can utilize or ferment feed in different ways and produce different metabolites (42, 43). PCA and PCoA analysis of cecal contents revealed a certain degree of diversity in cecal microbiota, similar outcomes were achieved in studies by the other researchers (6, 27).

Some studies have shown that fecal microbiota is related to the growth performance of broilers. Such as the study which has shown that compared with broilers with high FCR, broilers with low FCR have higher and lower abundances of *Firmicutes* and *Bacteroidetes*, respectively. In addition, *Firmicutes* in broiler feces are more abundant in a fat meat line than in a lean meat line, and the situation of *Bacteroidetes* is just the opposite (44–46). Meanwhile, a study showed that in the treatment group of enramycin and 3 g/kg *B. licheniformis*, the abundance of *Firmicutes* in the feces of heavier broilers was also higher (6). The *Firmicutes* and *Bacteroidetes* dominated the intestinal composition of Pekin ducks at all growth stages, which is consistent with previous studies of other animals (47, 48). At 21 d, approximately 90% of the relative abundant phyla were *Firmicutes* in the intestine of Pekin ducks fed a diet with 400 mg/kg *B. licheniformis*. This finding may be associated with those younger animals needing more intestinal bacteria members that belonged to *Firmicutes* for digestion and absorbance of nutrition, for instance, *Lachnospiraceae, Ruminococcaceae, Erysipelotrichaceae*, and *Streptococcaceae*. As Pekin ducks aged, the percentages of *Firmicutes* phyla were reduced, while the population of *Bacteroidetes* phyla was increased in the gut with continuous dietary *B. licheniformis*. These results are consistent with those previously reported. Many studies have confirmed the probiotic effects of *Bacteroides* and *Firmicutes*. They seem to play an important role in polysaccharide decomposition, so they help to improve nutrient utilization, promote the development of the immune system, and maintain an intestinal microecological balance (49, 50).

There are also quite a number of studies that reported that dietary probiotics have a positive effect in facilitating the abundance of beneficial bacteria and reducing the colonization of potential zoonotic pathogens in the gastrointestinal tract of broilers (1, 51). During the study, we can find the addition of *B. licheniformis* enriched bacteria were mostly anaerobes and SCFA-producing bacteria, including *Intestinimonas, Collinsella, Christensenellaceae, Lachnospiraceae, Anaerofilum*, and *Bilophila*. Among them, the genus *Lachnospira* may be a potentially beneficial bacterium, participating in the metabolism of a variety of carbohydrates, among which acetic acid, the fermentation product, is the main source of energy for the host. *Collinsella* mainly produces some gases in the intestine, which has been reported to be associated with abnormal lipid metabolism and type 2 diabetes. *Christensenellaceae* is significantly negatively associated with BMI and metabolic diseases such as inflammation, fat deposition, IBD, and metabolic syndrome. *Anaerotruncus* can participate in the glucose metabolism pathway, and the final metabolites are beneficial substances acetic acid and butyric acid. *Bilophila* thrives in the gut rich in bile acids, and a high-fat diet can boost its proportion of the gut flora, increasing the risk of inflammatory bowel disease and hepatobiliary disease (8, 52–57). These abundance shifts in bacteria were then supported by the increased gut microbiota functions of carbohydrate metabolism which are responsible for the gut microbial fermentation of carbohydrates under a strictly anaerobic environment to produce SCFAs (58).

A correlation analysis and significance test further demonstrated that the lipid transport and metabolism of intestinal microbiota in 400 mg/kg *B. licheniformis* supplementation group were significantly affected, specific performance in lipid transport, fatty acid degradation, and glycerol phospholipid metabolism. In fact, in the past decade, the potential role of intestinal microorganisms in the development of various diseases has attracted considerable attention. In particular, gut microbiota has been identified as a major risk factor for many metabolic disorders, such as obesity, type 2 diabetes, and non-alcoholic fatty liver disease. In recent years, intestinal microorganisms have become the focus of attention as the potential driving mechanism of obesity and its associated diseases. The gut microbiota is a key mediator in energy acquisition because it converts food into host nutrition, and obesity-related gut microbiota is more capable of acquiring energy from the diet (59). In obese individuals, the composition of microbial groups changed significantly, such as the contents of *Akkermansia, Faecalibacterium, Oscillibacter*, and *Alistipes* decreased significantly. Changes of serum metabolites related to intestinal microbial composition in obesity (56). Our results showed that 400 mg/kg *B. licheniformis* could improve the cecal microbial community structure of Pekin ducks and increase the abundance of intestinal microbiota related to SCFA metabolism, thus affecting the lipid transport and metabolism of the body.

## Conclusion

We found that adding 400 mg/kg *B. licheniformis* to the feed can improve the growth performance of Pekin ducks, and this beneficial effect may be due to the addition of *B. licheniformis* affecting the intestinal microbiota structure, improving the contents of SCFA-producing bacteria which can affect the lipid metabolism and transport. There are still calls for further investigation of how *B. licheniformis* affects lipid metabolism by regulating intestinal microbiota.

## Data Availability Statement

The original contributions presented in the study are included in the article/supplementary material, further inquiries can be directed to the corresponding author.

## Ethics Statement

The animal study was reviewed and approved by Guidelines for Care and Use of Laboratory Animals of China Agricultural University.

## Author Contributions

ZX: conceptualization and supervision. LL: data curation, microbial analysis, writing—original draft, and review and editing. XH, Xl, KA, and WG: assisted with the experiments. CS: assisted in the manuscript preparation. All authors contributed to the article and approved the submitted version.

## Conflict of Interest

The authors declare that the research was conducted in the absence of any commercial or financial relationships that could be construed as a potential conflict of interest.

## Publisher's Note

All claims expressed in this article are solely those of the authors and do not necessarily represent those of their affiliated organizations, or those of the publisher, the editors and the reviewers. Any product that may be evaluated in this article, or claim that may be made by its manufacturer, is not guaranteed or endorsed by the publisher.
